# Long non-coding RNA FGD5-AS1 promotes non-small cell lung cancer cell proliferation through sponging hsa-miR-107 to up-regulate FGFRL1

**DOI:** 10.1042/BSR20193309

**Published:** 2020-01-24

**Authors:** Yafeng Fan, Hongxia Li, Zhongping Yu, Wen Dong, Xiaoyan Cui, Jinlian Ma, Shengwen Li

**Affiliations:** 1 Department of Respiratory, Shanxi Cancer Hospital, Taiyuan 030013, Shanxi, China; 2 Department of Oncology, Shanxi Provincial People’s Hospital, Taiyuan 030012, Shanxi, China; 3 Department of Oncology, Shouyang Second People’s Hospital, Jinzhong 030600, Shanxi, China; 4 Shouyang Hospital of Traditional Chinese Medicine, Jinzhong 030600, Shanxi, China

**Keywords:** FGFRL1, hsa-miR-107, LncRNA FGD5-AS1, non-small cell lung carcinoma

## Abstract

Long non-coding RNA (lncRNA) FYVE, RhoGEF and PH domain containing 5 antisense RNA 1 (FGD5-AS1) has been reported as an oncogene in colorectal cancer, promoting its tumorgenesis. The present paper focused on searching the potential function of FGD5-AS1 in non-small cell lung carcinoma (NSCLC). There are connections between the expression of lncRNA FGD5-AS1 and human NSCLC tumor growth and progression. Also, the relationships between FGD5-AS1, hsa-miR-107 and mRNA fibroblast growth factor receptor like 1 (FGFRL1) are going to test their interaction in NSCLC cell lines, which may cause a series of biological behaviors of NSCLC cells. qRT-PCR analysis was conducted to test the expression of RNAs in different situation. CCK-8 experiment and clone formation assay were performed to assess proliferation of NSCLC cells. Also, connection between FGD5-AS1 and hsa-miR-107 were investigated by luciferase reporter assay and RNA pull-down assay. Rescue experiments were performed to verify the modulating relationship between FGD5-AS1, hsa-miR-107 and FGFRL1. High-level expression of FGD5-AS1 was found in NSCLC. FGD5-AS1 may promote the proliferation of NSCLC cells. Also, the combination between hsa-miR-107, FGD5-AS1 and NSCLC have been proved, which means they can play an interaction function in NSCLC cells. Thence, we concluded that lncRNA FGD5-AS1 promotes non-small cell lung cancer cell proliferation through sponging hsa-miR-107 to up-regulate FGFRL1.

## Introduction

The situation of death caused by cancer is appalling, and one of the most malignant cancers is lung cancer [1]. Non-small cell lung cancer is most deaths causing cancer, which accounts for approximately 80% of all lung cancers, and approximately 75% of patients are found to be in the advanced stage, resulting in a low 5-year survival rate. There are some examples to prove this fact. First of all, the 5-year survival rate of non-small cell lung carcinoma (NSCLC) is only 11%, which means most patients can not get completely cured after their therapy [2]. Second, tumor invasion and metastasis are the major problems in disease progression and therapy failure, which cause immense cases of decease [3]. Thence, a strategy of prevention from tumor metastasis is really essential.

MiRNAs are a group of small non-coding RNAs which consist of 20-24 nucleotides. They are found to adjust target mRNAs’ expression by binding 3-untranslated region of mRNAS [4]. And miRNAs participate in most cellular functions, such as proliferation, apoptosis, differentiation and metastasis [5]. MiRNAs are important genes in modulating cancer, such as suppressors or oncogenes, for their aberrant expression and dysregulation can affect cancer cell greatly [6,7]. MiR-107 was being found as a tumor suppressor gene in colorectal cancer [8]. This is because many cancer-specific signaling pathways are affected by miR-107’s oncogenic expression [9–13]. Once miR-107 expression rise, it will cause the apoptosis of CRC cell lines [9]. As people have learned much about miR-107’s tumor suppressor character, we need to dig more about its function in NSCLC.

As a type of single-stranded RNA, messenger RNA is transcribed from one strand of DNA as a template and carries genetic information to direct protein synthesis [14]. Fibroblast growth factor receptor like 1 (FGFRL1) is an mRNA which can cause oncogenous possibility of cells in esophageal squamous cell carcinomas [15]. Meanwhile, FGFRL1 is modulated by hsa-miR-107, and much evidence suggest that miR-107 has the function as tumor suppressor, which has been found that miR-107 has been down-regulated in human colorectal cancer cell lines and it can suppress the proliferation, migration and invasion of colorectal cancer cells [8].

Long non-coding RNA (LncRNA) FYVE, RhoGEF and PH domain containing 5 antisense RNA 1 (FGD5-AS1) has been reported as oncogene in colorectal cancer, promoting its tumorigenesis [16]. Mechanistically, lncRNAs can exert functions in human cancers by sequestering miRNAs to up-regulate downstream protein-coding mRNAs [17–19]. According to previous study, we can speculate that FGD5-AS1 has its critical role in tumor progression, and we found that it can sponge to miR-107 in our assays. Also, according to our experiments, we found that FGD5-AS1 plays a critical role in NSCLC proliferation. In the present study, the expression of FGD5-AS1 in NSCLC tissues was investigated and we found that FGD5-AS1 was overexpressed in NSCLC cell lines. Moreover, qRT-PCR and luciferase reporter assays suggested that hsa-miR-107 targets FGFRL1, and rescue experiment showed there are connections between FGD5-AS1, hsa-miR-107 and FGFRL1. In the present study, we aimed to indicate that FGD5-AS1 may promote non-small cell lung cancer cell proliferation through sponging hsa-miR-107 to up-regulate FGFRL1. And this work may provide a new train of thought for speculating the function of FGD5-AS1 in NSCLC.

## Materials and methods

### Tissue samples

Fifty NSCLC tissues and adjacent non-tumor tissues were collected at Shanxi Provincial People’s Hospital from April 2014 to January 2018. All the patients did not accept any treatment before the present study. The study has been approved by the Ethics Committee of Shanxi Provincial People’s Hospital and informed consent was signed by participants. Besides, fresh tissues were frozen in liquid nitrogen and preserved at −80°C.

### Cell culture

Human embryonic lung fibroblast cell (IMR-90) and NSCLC cells (NCI-H1703, NCI-H1793, NCI-H1869) were bought from Chinese Academy of Sciences (Beijing, China) and cultured in RPMI-1640 medium (Invitrogen, Carlsbad, CA, U.S.A.) containing 10% fetal bovine serum (FBS; Invitrogen) and 1% penicillin/streptomycin (Sigma–Aldrich, Milan, Italy). All cells were cultured in an incubator with 5% CO_2_ at 37 °C.

### Cell transfection

Specific shRNAs against FGD5-AS1 (sh-FGD5-AS1#1#2) and the negative control (shNC) were gained from GenePharma (Shanghai, China). The pcDNA3.1/FGFRL1 and the empty pcDNA3.1 vector (GenePharma) were used for overexpression studies. The miR-107 mimics and NC mimics were obtained from GenePharma. And these plasmids were transfected into NCI-H1703 and NCI-H1793 cells by Lipofectamine 2000 (Invitrogen). Sequences for above plasmids were:

- sh-NC: 5′-CCGGTTGAAAAAAGGGGGAAAAAAACTCGAGTTTTTTTCCCCCTTTTTTCAATTTTTG-3′
- sh-FGD5-AS1#1: 5′-CCGGCACTTGATATTAGTAATTTGACTCGAGTCAAATTACTAATATCAAGTGTTTTTG-3′
- sh-FGD5-AS1#2: 5′-CCGGGGCATGGTAAAAGAGTTAACTCTCGAGAGTTAACTCTTTTACCATGCCTTTTTG-3′
- NC-mimics: 5′-ACGUCGAAUGUACAGGGCUAUCA-3′
- hsa-miR-107 mimics: 5′-AGCAGCAUUGUACAGGGCUAUCA-3′

Sequences for FGFRL1 overexpression are provided in Supplementary Table S1.

### qRT-PCR

TRIzol reagent (Invitrogen) was employed for extracting complete RNA. The total RNA was reverse-transcribed into cDNA using Reverse Transcription Kit (Takara, Kusatsu, Japan). And RT-qPCR analysis was progressed via SYBR Green Premix PCR Master Mix (Roche, Mannheim, Germany) under ABI HT9600 (Applied Biosystems, Foster City, CA, U.S.A.). Fold expression changes were calculated by utilization of 2^−ΔΔ*C*_t_^ method and *GAPDH/U6* was shown as endogenous gene. The primers for PCR were listed as below:

- FGD5-AS1: Forward: 5′-CGTGGAGAAGAATTGGGC-3′, Reverse: 5′-CGTGGAGAAGAATTGGGC-3′
- miR-107: Forward: 5′-AGCAGCATTGTACAGGGCTATC-3′, Reverse: 5′-CTCTACAGCTATATTGCCAGCCAC-3′
- miR-103a-3p: Forward: 5′-AGCAGCATTGTACAGGGCTATG-3′, Reverse: 5′-CTCTACAGCTATATTGCCAGCCAC-3′
- miR-6838-5p: Forward: 5′-AAGCAGCAGTGGCAAGACTC-3′, Reverse: 5′-CTCTACAGCTATATTGCCAGCCAC-3′
- FGFRL1: Forward: 5′-GTGTGAGGAGCATGGGTCTC-3′, Reverse: 5′-GTGTGTGTGTGTGTGTGTGGA-3′
- RAB11FIP2: Forward: 5′-AGGGCTATCAGATCCCCCA-3′, Reverse: 5′-AAGCAGCGAAACATGGGAC-3′
- RDH11: Forward: 5′-AGCAGGTGTTGGTGCGGAAACT-3′, Reverse: 5′-CGGACACATCATCACTCCTGCA-3′
- GAPDH: Forward: 5′-ACCTGACCTGCCGTCTAGAA-3′, Reverse: 5′-GTCAAAGGTGGAGGAGTGGG-3′
- U6: Forward: 5′-CTCGCTTCGGCAGCACA-3′, Reverse: 5′-AACGCTTCACGAATTTGCGT-3′

### CCK-8 assay

Cells (1 × 10^3^) were inoculated in 96-well plates and cultured over specific time points. Ten microliters of CCK-8 reagent were added into each well and incubated for another 4 h. Absorbance at 450 nm was examined by using a microplate reader (Bio-Tek Instruments, Hopkinton, MA, U.S.A.).

### Colony formation assay

Transfected NCI-H1703 and NCI-H1793 cells (1 × 10^3^) were seeded into fresh six-well plates. After incubating for 2 weeks, the cells were fixed and dyed in methanol (Sigma–Aldrich) and Crystal Violet solution (Sigma–Aldrich). Colonies were counted manually.

### Apoptosis assay

The apoptosis of NCI-H1703 and NCI-H1793 cells were cultured in six-well plates for 48 h. Then, cells were fixed with ice-cold 70% ethanol (Sigma–Aldrich) at 4°C for 2 h and rinsed with PBS (Solarbio, Beijing, China). Moreover, cells were stained utilizing Annexin V-fluorescein isothiocyanate and propidium iodide (PI). Finally, apoptosis rates were estimated using Flow cytometry (Becton Dickinson, Franklin Lakes, NJ, U.S.A.).

### TUNEL assay

TUNEL experiment was conducted via *In Situ* Cell Death Detection Kit (Roche, Basel, Switzerland). Cell suspension was applied on the coverslip, then 4% paraformaldehyde (Sigma–Aldrich) was added for fixing cells for 1 h. Afterward, NCI-H1703 and NCI-H1793 cells were then treated with 0.1% Triton X-100 for 3 min, and TUNEL solution was added later. Following nuclei staining with 4′,6-diamino-2-2phenylindole (DAPI), fluorescence intensity was determined through the fluorescence microscope (Olympus, Tokyo, Japan).

### Subcellular fractionation

The isolation of nuclear and cytosolic fractions was completed using the PARIS Kit (Life Technologies, Carlsbad, CA, U.S.A.). Relative expression level of FGD5-AS1 was evaluated by qRT-PCR. And GAPDH or U6 was cytoplasmic control or nuclear control, respectively.

### Western blot

Total protein was obtained from cells which were lysed by RIPA lysis buffer adding with protease inhibitors. Then protein extracts were separated by using SDS/PAGE and moved to PVDF membranes (General Electric Healthcare, Buckinghamshire, U.K.). After being blocked with fat-free milk, proteins were co-incubated with primary antibodies for FGFRL1 (ab95940, Abcam, Cambridge, U.S.A.) and GAPDH (ab8245, Abcam) overnight. Furthermore, secondary antibodies were added for cultivating for 1 h. Chemiluminescence detection system was used for detecting the amount of protein.

### Luciferase reporter assay

The wild-type (WT) and mutant (MUT) binding sites of miR-107 in FGD5-AS1 sequence or FGFRL1 3′UTR was subcloned into pmirGLO dual-luciferase vector to construct FGD5-AS1-WT/MUT or FGFRL1-WT/MUT. And then plasmids were treated with indicated transfection plasmids into NCI-H1703 and NCI-H1793 cells, severally. The luciferase activity was examined via Dual-Luciferase Reporter Assay System (Promega, MA, U.S.A.).

### RNA binding protein immunoprecipitation assay

RNA binding protein immunoprecipitation (RIP) experiment was carried out by the utilization of Magna RIP™ RNABinding Protein Immunoprecipitation Kit (Millipore). NCI-H1703 and NCI-H1793 cells were lysed in RIP lysis buffer, then cultured with magnetic beads conjugated with human anti-argonaute RISC catalytic component 2 (Ago2) or with a negative control anti-IgG antibody. The qRT-PCR was performed to evaluate the expression level of FGD5-AS1.

### RNA pull-down assay

The miR-107-Wt, miR-107-Mut and NC were biotin-labeled into Bio-miR-107-Wt, Bio-miR-107-Mut and Bio-NC. Streptavidin-coated magnetic beads (Life Technologies) were applied for incubation with cell lysates and biotinylated RNAs. Pull-down assay was completed in biotin-coupled RNA complex. The abundance of FGD5-AS1 in bound fractions was determined by RT-qPCR.

### Ethynyl deoxyuridine incorporation assay

Transfected NCI-H1703 and NCI-H1793 was placed into 12-well plate for incubation, and cells were then incubated with 300 μl Ethynyl deoxyuridine (EdU) labeling media for 2 h. After that, 4% paraformaldehyde and 0.5% Triton X-100 were used for immobilization of cells, and cells were reacted with Apollo reaction liquid for 30 min. Hoechst33342 was utilized for nuclear counterstain. The EdU-positive cells were captured under a fluorescence microscope (Olympus).

### Statistical analysis

All experiments were run thrice independently. Data were manifested as means ± SD. Overall survival was analyzed by Kaplan–Meier method. One-way analysis of variance (ANOVA) was used to confirm the difference of multiple groups and Student’s *t* test was employed for two groups. Statistical analysis was carried out by SPSS software (version 19.0; SPSS, Chicago, IL, U.S.A.). Gene expression correlation analysis was performed with Pearson’s method.

## Results

### LncRNA FGD5-AS1 was highly expressed in NSCLC tissues and cells, and FGD5-AS1 promoted NSCLC cells’ proliferation

**Figure 1:**
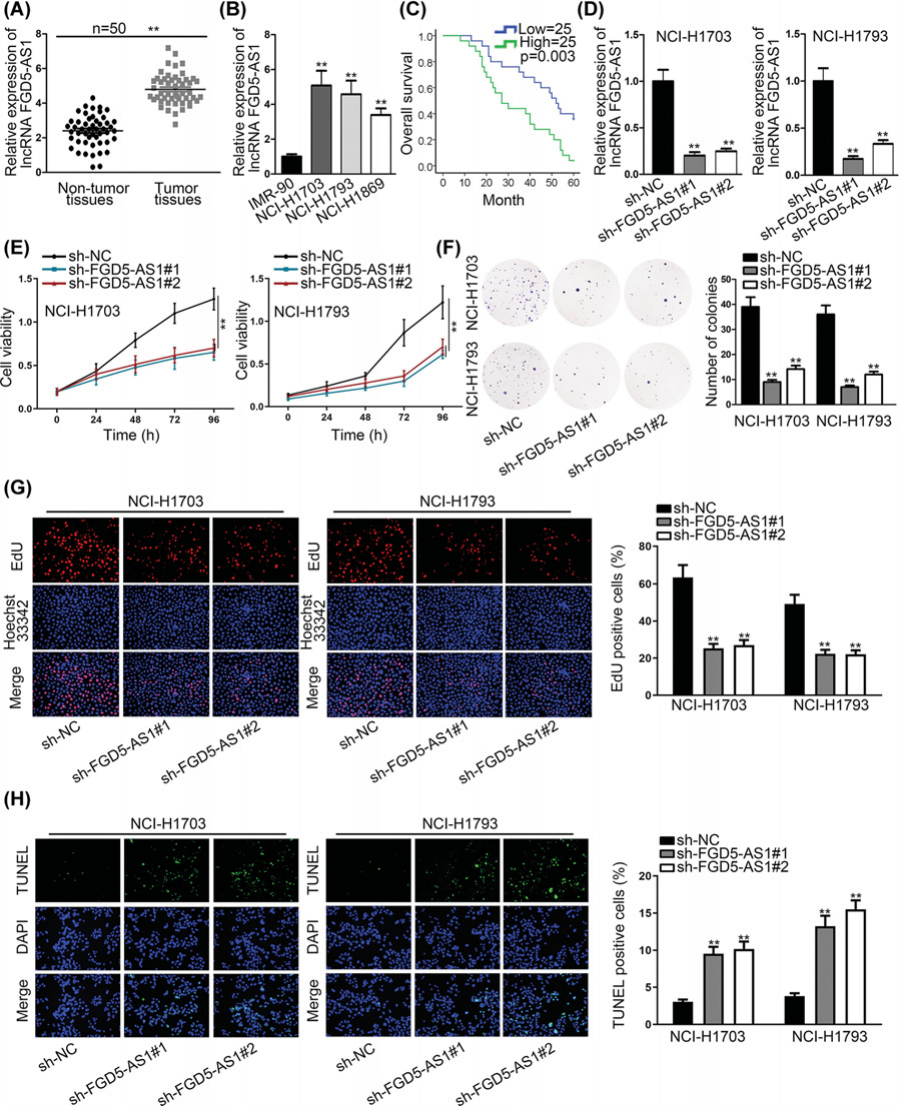
LncRNA FGD5-AS1 was highly expressed in NSCLC tissues and cells, and promoted the proliferation of NSCLC cells (**A**) qRT-PCR assays were performed to investigate the expressions of FGD5-AS1 in NSCLC tumor tissues and non-tumor tissues. (**B**) The expressions of FGD5-AS1 were detected in cell lines (IMR-90, NCI-H1703, NCI-H1793 and NCI-H1869) by qRT-PCR assays. (**C**) The survival rate of high expression and low expression of FGD5-AS1 patient groups were contrasted by Pearson’s experiment. (**D**) qRT-PCR assays were conducted to detect the inference efficiency of FGD5-AS1 in two different NSCLC cell lines, NCI-H1703 and NCI-H1793. (**E**–**G**) CCK-8, colony formation and EdU assays were implemented to detect the relative cell proliferation ability in NSCLC cell lines (NCI-H1703 and NCI-H1793) by sh-FGD5-AS1#1, #2. (**H**) TUNEL was performed to appraise the apoptosis rate of two NSCLC cell lines (NCI-H1703 and NCI-H1793). **P<0.01 indicated that differences between groups were statistically significant.

In the beginning, expression of FGD5-AS1 in non-small cell lung cancer tissue cells was investigated. According to qRT-PCR analysis, higher expression level of FGD5-AS1 in NSCLC tissue and cells was found than non-tumor tissue and cells in control (Figure 1A,B). Overall survival of NSCLC patients according to high and low expression of FGD5-AS1 was detected in Figure 1C, and we found the patients with high expression of FGD5-AS1 have a relative lower overall survival than the patients with low expression of FGD5-AS1. Further, we also discovered a close association between high FGD5-AS1 expression and larger tumor size (Table 1). And then, to investigate the precise function of FGD5-AS1 in NSCLC, we carried out loss-of-function assays after confirming the inhibition of FGD5-AS1 in NCI-1703 and NCI-1793 cells by qRT-PCR analysis in Figure 1D. Also, CCK-8 experiment and clone formation assay were performed to assess the proliferation of NSCLC cell according to different group of interfered FGD5-AS1 (Figure 1E,F). The inhibition of cell proliferation induced by silenced FGD5-AS1 was further confirmed according to the results of EdU assay (Figure 1G). And we found that relative cell viability and number of colonies in interfered FGD5-AS1 group were much lower than negative control group, which meant that FGD5-AS1 affects the proliferation of NSCLC cells significantly. Flow cytometry analysis and TUNEL were performed to analyze the apoptosis of NSCLC cell according to different group of interfered FGD5-AS1 (Figure 1H and Supplementary Figure S1A). We found that interfered FGD5-AS1 caused much more NSCLC cell apoptosis than control group. Thence, according to the result we can conclude that the expression of FGD5-AS1 has a strong association with NSCLC proliferation, which is that higher expression of FGD5-AS1 promotes more proliferation of NSCLC cells, and FGD5-AS1 can act as an oncogene in NSCLC cells.

**Table 1:** Correlation between FGD5-AS1 expression and clinical features

Variable	FGD5-AS1 expression		*P*-value
	Low	High	
**Sex**			
Male	12	15	0.571
Female	13	10	
**Age**			
≤60	14	16	0.773
>60	11	9	
**Histological grade**			
Middle or low	13	11	0.778
High	12	14	
**TNM stage**			
I/II	14	3	0.002*
III/IV	11	22	
**Lymph node metastasis**			
Negative	6	8	0.754
Positive	19	17	
**Tumor size**			
≤3 cm	17	6	0.004*
>3 cm	8	19	
**History of smoking**			
Never	7	10	0.551
Ever	18	15	

(*n*=50). Low/high by the sample median. Pearson’s χ^2^ test.

*P<0.01 was considered to be statistically significant.

### Hsa-miR-107 was lowly expressed in NSCLC tissues and cells, and FGD5-AS1 could bind with hsa-miR-107

**Figure 2:**
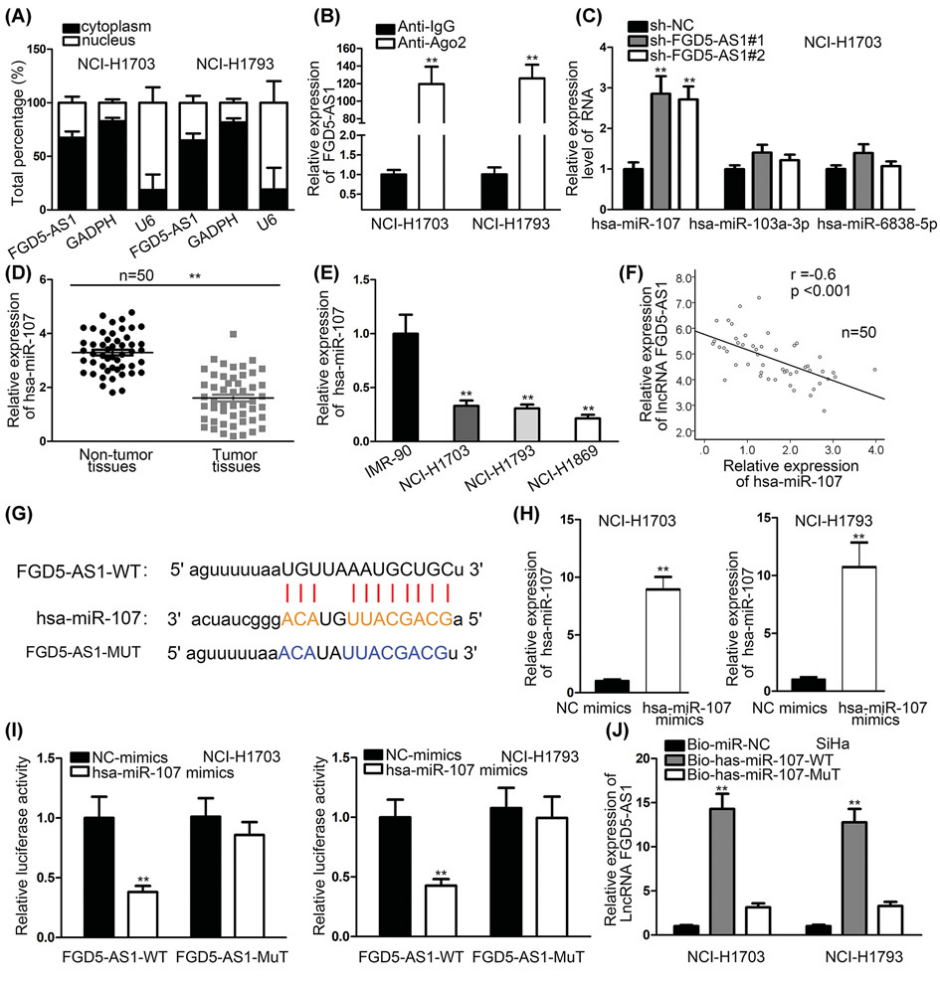
LncRNA FGD5-AS1 can bind to hsa-miR-107 (**A**) Nuclear–cytoplasmic fractionation was implemented to prove FGD5-AS1 was in cytoplasm. (**B**) FGD5-AS1 enrichment in RISC was proved by RIP assay. (**C**) A contrast of the expression of hsa-miR-107 and other two miRNAs (hsa-miR-103a-3p and hsa-miR-6838-5p) was made by qRT-PCR analysis to find the most proper miRNA. (**D**) qRT-PCR analysis investigated the relative expression of hsa-miR-107 in NSCLC tissues and in normal tissues. (**E**) The expression of hsa-miR-107 was detected in cell lines (IMR-90, NCI-H1703, NCI-H1793 and NCI-H1869) by qRT-PCR assays. (**F**) The negative correlation in hsa-miR-107 and FGD5-AS1 was conducted by Kaplan–Meier method. (**G**) Binding sites between FGD5-AS1 and hsa-miR-107 were presented by bioinformatics. (**H**) qRT-PCR assays were used to detect the overexpression efficiency of hsa-miR-107 in NSCLC cell lines (NCI-H1703 and NCI-H1793). (**I,J**) RNA pull-down assays and luciferase reporter assays were performed to detect the binding relationship between FGD5-AS1 and hsa-miR-107 in NSCLC cell lines (NCI-H1703 and NCI-H1793). **P<0.01, ***P<0.001 indicated statistically significant differences in this figure.

Second, connection between FGD5-AS1 and hsa-miR-107 were investigated to explore their combination. At first, we detected that FGD5-AS1 mainly stayed in cytoplasm using Subcellular Fraction assay, and RIP assay proved that FGD5-AS1 was abundantly enriched in RNA induced silenced complex (RISC). (Figure 2A,B). Meanwhile, a contrast of the expression of hsa-miR-107 and other two miRNAs (hsa-miR-103a-3p and hsa-miR-6838-5p) under the condition of FGD5-AS1 depletion was made by qRT-PCR analysis to find the most proper miRNA (Figure 2C). The result showed that hsa-miR-107 is the most suitable one. The expression level of hsa-miR-103a-3p and hsa-miR-6838-5p in NSCLC cells and normal control cell presented no significant difference (Supplementary Figure S1B). Meanwhile, we used qRT-PCR analysis to detect the relative expression of hsa-miR-107 in NSCLC cells and tumor tissues, and we can see that relative expression of hsa-miR-107 in NSCLC cells and tissues is much lower than normal cells and tissues (Figure 2D,E). Moreover, the negative correlation between hsa-miR-107 and FGD5-AS1 was investigated by Pearson’s experiment in Figure 2F. According to bioinformatics, we drew the binding site of hsa-miR-107 and FGD5-AS1 (Figure 2G). Overexpression effect of hsa-miR-107 was detected by qRT-PCR analysis in Figure 2H. Luciferase reporter assay and RNA Pull down assay were implemented to examine the connection between FGD5-AS1 and hsa-miR-107, which indicated that FGD5-AS1 and hsa-miR-107 have a binding relationship (Figure 2I,J). Therefore, a conclusion can be drawn that FGD5-AS1 and hsa-miR-107 can combine with each other.

### FGFRL1 was highly expressed in NSCLC tissues and cells, and hsa-miR-107 can target FGFRL1

**Figure 3:**
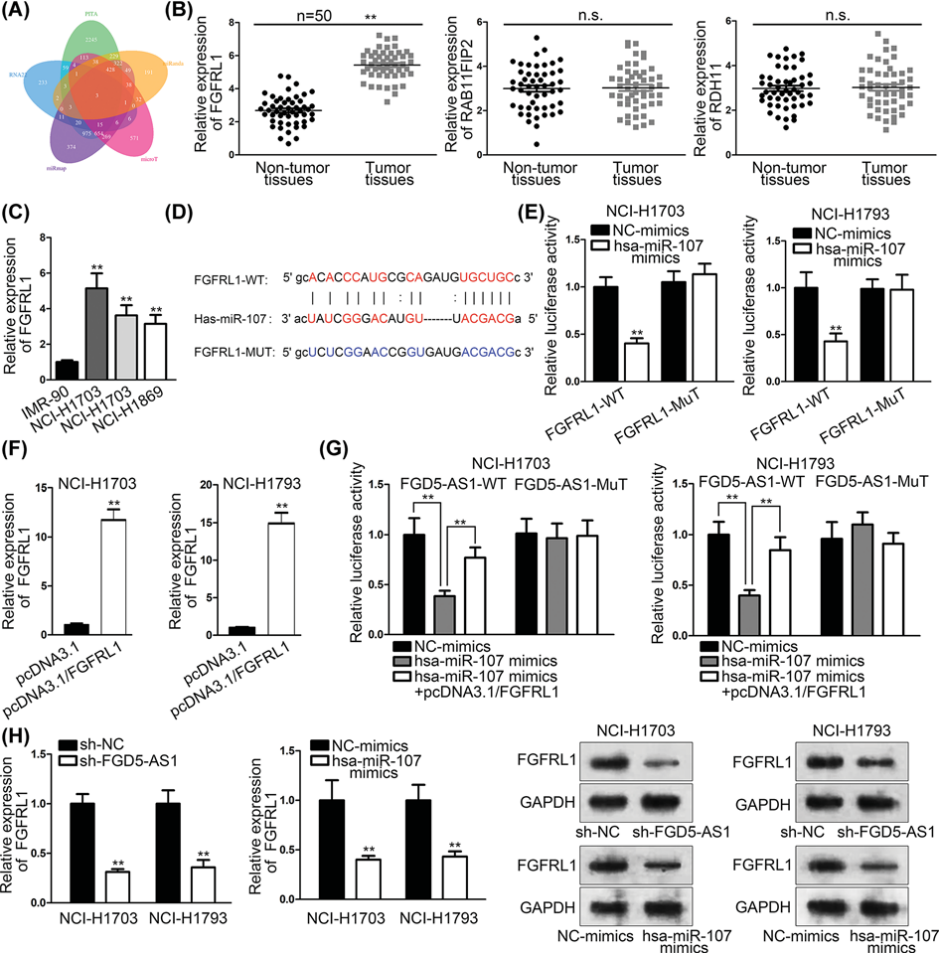
Hsa-miR-107 targeted FGFRL1 (**A**) StarBase was used to predict potential mRNA. (**B**) The expressions of three different types of mRNA were detected by qRT-PCR assays. (**C**) qRT-PCR assays were used to detect the expressions of FGFRL1 in cell lines (IMR-90, NCI-H1703, NCI-H1793 and NCI-H1869). (**D**) Binding sites between FGFRL1 and hsa-miR-107 were presented by bioinformatics. (**E**) Luciferase reporter assay was used to test the binding capacity of FGFRL1 and hsa-miR-107. (**F**) Overexpression efficiency of FGFRL1 was explored by qRT-PCR assays. (**G**) Luciferase reporter assay implemented to test the luciferase activity of wild and mutant FGD5-AS1. (**H**) Western blot and qRT-PCR assays were used to verify that interfered FGD5-AS1 and hsa-miR-107 can affect the expression of FGFRL1. **P<0.01 indicated statistically significant differences in this figure which n.s. indicated no significance.

Then, StarBase was used to probe an mRNA as target gene (Figure 3A), and we found three of them (FGFRL1, RAB11FIP2 and RDH11) through intersection of five bioinformatics tools. qRT-PCR assays were used to investigate which mRNA is the most active one targeting hsa-miR-107, and results showed that FGFRL1 is the most suitable one (Figure 3B). Then, qRT-PCR assays were used to detect the expressions of FGFRL1 in cell lines (IMR-90, NCI-H1703, NCI-H1793 and NCI-H1869) (Figure 3C). Binding sites between FGFRL1 and hsa-miR-107 were presented by bioinformatics (Figure 3D). We tested the connection between hsa-miR-107 and FGFRL1, using luciferase reporter assay, and the result showed that hsa-miR-107 and FGFRL1 can bind to each other (Figure 3E). Overexpression efficiency of FGFRL1 was explored by qRT-PCR assays (Figure 3F). Luciferase reporter assay was implemented, and the result indicated that FGFRL1 rescued the suppressive effects of miR-107 on luciferase activity of wild FGD5-AS1 (Figure 3G). Western blot and qRT-PCR assays were used to verify whether interfered FGD5-AS1 and hsa-miR-107 can affect the expression of FGFRL1 or not, and the results indicated that FGD5-AS1 and hsa-miR-107 can affect the expression of FGFRL1 (Figure 3H). Thence, we can conclude that FGFRL1 was high expressed in NSCLC tissues and cells, and hsa-miR-107 could bind with FGFRL1. Also, interfered FGD5-AS1 and hsa-miR-107 can affect the expression of FGFRL1.

### Rescue assay approved the interaction among FGD5-AS1, hsa-miR-107 and FGFRL1

**Figure 4:**
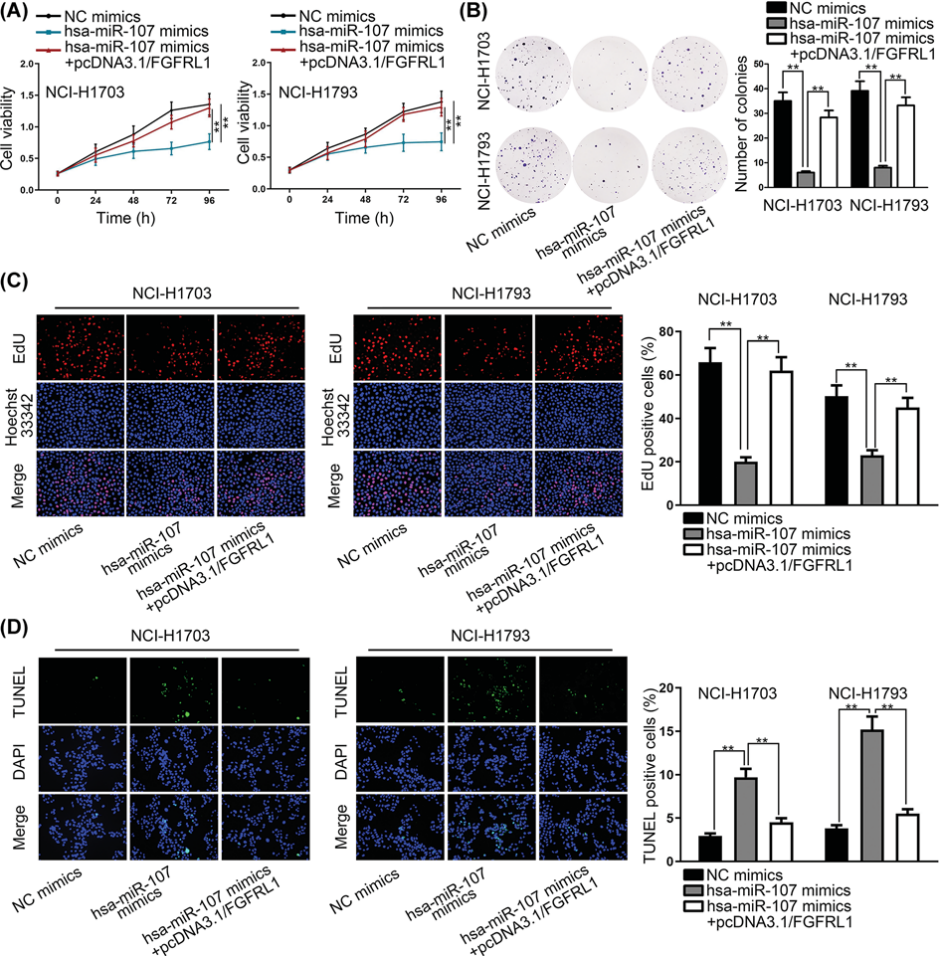
Rescue experiment between FGFRL1 and hsa-miR-107 (**A–C**) CCK-8, EdU and colony formation assays were performed to prove that the decreased proliferation ability of NSCLC cells by hsa-miR-107 mimics was offset by hsa-miR-107 mimics + pcDNA3.1/FGFRL1. (**D**) TUNEL indicated that the rate of NSCLC cells’ apoptosis increased by hsa-miR-107 mimics was recovered by hsa-miR-107 mimics+pcDNA3.1/FGFRL1. **P<0.01 indicated statistically significant differences in this figure.

**Figure 5:**
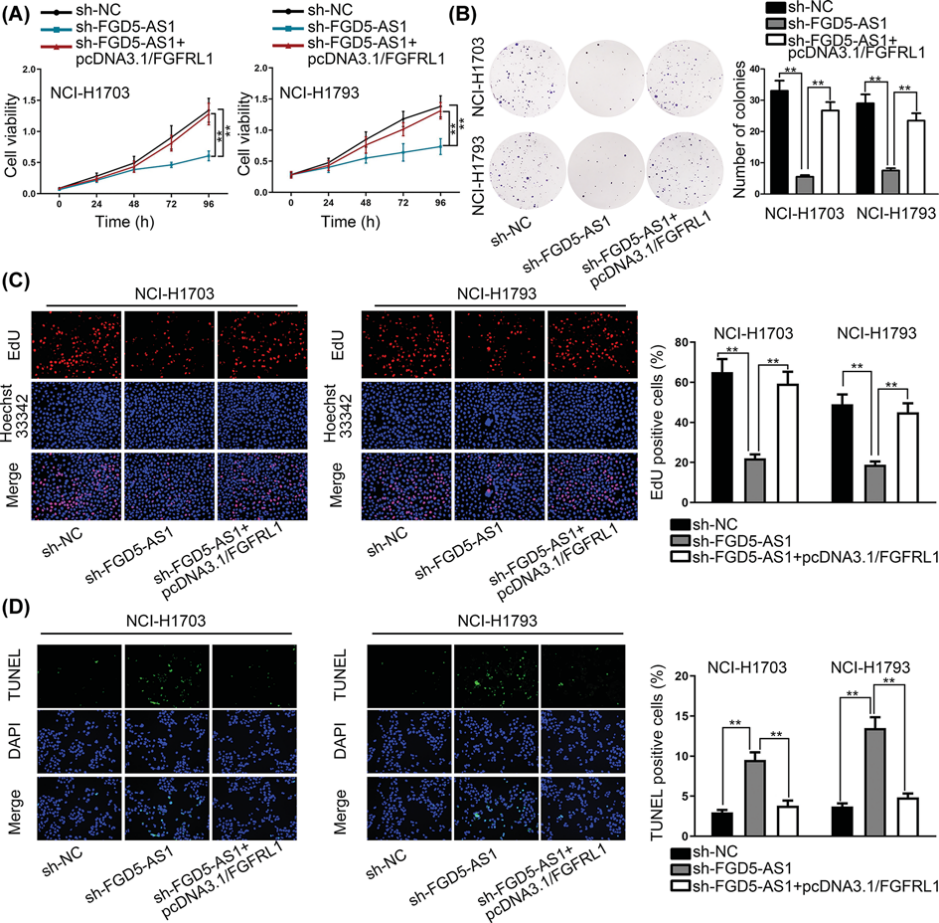
Rescue experiment between FGD5-AS1 and FGFRL1 (**A**–**C**) CCK-8, colony formation and EdU assays were performed to prove that the decreased proliferation ability of NSCLC cells by sh-FGD5-AS1 was offset by sh-FGD5-AS1+pcDNA3.1/FGFRL1. (**D**) TUNEL indicated that the rate of NSCLC cells apoptosis increased by sh-FGD5-AS1 was recovered by sh-FGD5-AS1+pcDNA3.1/FGFRL1. **P<0.01 indicated statistically significant differences in this figure.

Rescue experiments were performed to verify the modulating relationship between FGD5-AS1, hsa-miR-107 and FGFRL1 (Figures 4 and 5). Two rescue experiments were implemented, and we used CCK-8 assay, colony formation assay, EdU incorporation assay and TUNEL respectively to investigate the modulating relationship between FGD5-AS1, hsa-miR-107 and FGFRL1. To be specific, CCK-8, EdU and colony formation assays were performed, and the results showed that the decreased proliferation ability of NSCLC cells by hsa-miR-107 mimics was offset by hsa-miR-107 mimics+pcDNA3.1/FGFRL1. TUNEL proved that the rate of NSCLC cells’ apoptosis increased by hsa-miR-107 mimics was recovered by hsa-miR-107 mimics+pcDNA3.1/FGFRL1. As the same, CCK-8, EdU, colony formation assays and TUNEL were conducted again, but controlled groups were changed into sh-NC, sh-FGD5-AS1 and sh-FGD5-AS1+pcDNA3.1/FGFRL1. And the results vindicated the rescue relationship among these three RNAs, which proved the interactive relationship between FGD5-AS1, hsa-miR-107 and FGFRL1.

## Discussion

The expression of miRNAs is associated with various human cancers, which indicates that there might be methods for us to make miRNAs as therapeutic targets for cancer therapy because miRNAs can modulate LncRNA and mRNA’s gene expression [20]. Also, miRNAs’ dysregulation can interrupt the inner balanced RNA networks, so, it may affect the progression of cancer [21]. Therefore, miRNAs play a really important role in cells, and the study on them may give us more novel thoughts on the knowledge of molecular mechanisms on tumor.

LncRNA FGD5-AS1 has been reported as carcinogenic gene in colorectal cancer, which promotes the proliferation, migration, and invasion of colorectal cancer cells. In the present study, FGD5-AS1’s up-regulation in tumor tissues was relatively much higher than normal tissues. Moreover, in the present study it has been seen that higher level expression of FGD5-AS1 was associated with NSCLC. According to the experiments implemented in two NSCLC cell lines, we can conclude that FGD5-AS1 may promote the proliferation of NSCLC cells. However, the inner molecular mechanism of FGD5-AS1 in NSCLC cell is still unclear.

MiRNA sponge is a crucial role of lncRNAs. Recent years, increasing studies have revealed that lncRNAs can regulate tumor progression by sequestering miRNAs thus releasing their downstream target mRNAs and prompting mRNAs to exert functions [22–24]. Here, we applied bioinformatics analysis to screen out the miRNAs that potentially interact with FGD5-AS1. MiR-107 has been confirmed that it can play its tumor suppressor function in colorectal cancer [9], and hsa-miR-107 has low expression in NSCLC tumor tissues, such as NCI-H1703 and NCI-H1793. So, hsa-miR-107 may act as a tumor suppressor in NSCLC cells. Also, the binding relationship among hsa-miR-107, FGD5-AS1 and NSCLC was proved, thence they can play interaction function in NSCLC cells.

Rescue experiments found that FGD5-AS1 can modulate FGFRL1 and hsa-miR-107. Regarding the combined relationship of FGFRL1 and hsa-miR-107, we can see the importance of the expression of FGD5-AS1 in NSCLC cell. From all above, we can draw the conclusion that lncRNA FGD5-AS1 promotes non-small cell lung cancer cell proliferation through sponging hsa-miR-107 to up-regulate FGFRL1. Thus, these data revealed a novel ceRNA molecular pathway in NSCLC. This pathway might be a contributor to the progression of NSCLC. Lack of *in vivo* experiment and clinical analysis restricts the deep investigation of current study. We will focus on clinical significance of this novel pathway in our future study. Moreover, investigation on the upstream molecular mechanism of FGD5-AS1 will be the expansion of our next research.

## Conclusion

LncRNA FGD5-AS1 promotes non-small cell lung cancer cell proliferation through sponging hsa-miR-107 to up-regulate FGFRL1.

## Supplementary Material

Supplementary Figure S1-S2 and Table S1

## Abbreviations

CCK-8: cell counting kit-8
ceRNA: competing endogenous RNA
DAPI: 4′,6-diamino-2-2-phenylindole
EdU: ethynyl deoxyuridine
FBS: fetal bovine serum
FGD5-AS1: lncRNA FYVE, RhoGEF and PH domain containing 5 antisense RNA 1
FGFRL1: fibroblast growth factor receptor like 1
GAPDH: glyceraldehyde-3-phosphate dehydrogenase
IgG: immunoglobulin G
lncRNA: long non-coding RNA
miRNA: microRNA
MUT: mutant
NC: negative control
NSCLC: non-small cell lung carcinoma
PH: pleckstrin homology
PVDF: polyvinylidene fluoride
qRT-PCR: quantitative real-time polymerase chain reaction
RhoGEF: Rho guanine-nucleotide exchange factors
RIPA: radio immunoprecipitation assay
RIP: RNA binding protein immunoprecipitation
RISC: RNA-induced silencing complext
SD: standard deviation
SDS/PAGE: sodium dodecyl sulfate/polyacrylamide gel electrophoresis
TUNEL: terminal deoxynucleotidyl transferase dUTP nick end labeling
WT: wild-type
