# Supplementary material for: Long non-coding RNA FGD5-AS1 promotes non-small cell lung cancer cell proliferation through sponging hsa-miR-107 to up-regulate FGFRL1
Source: Biosci Rep. 2020 Jan 24;40(1):BSR20193309. doi: 10.1042/BSR20193309 (PMC6981095; doi:10.1042/BSR20193309)
Supplement: Supplementary Figure S1-S2 and Table S1 [file BSR-2019-3309_supp.pdf]

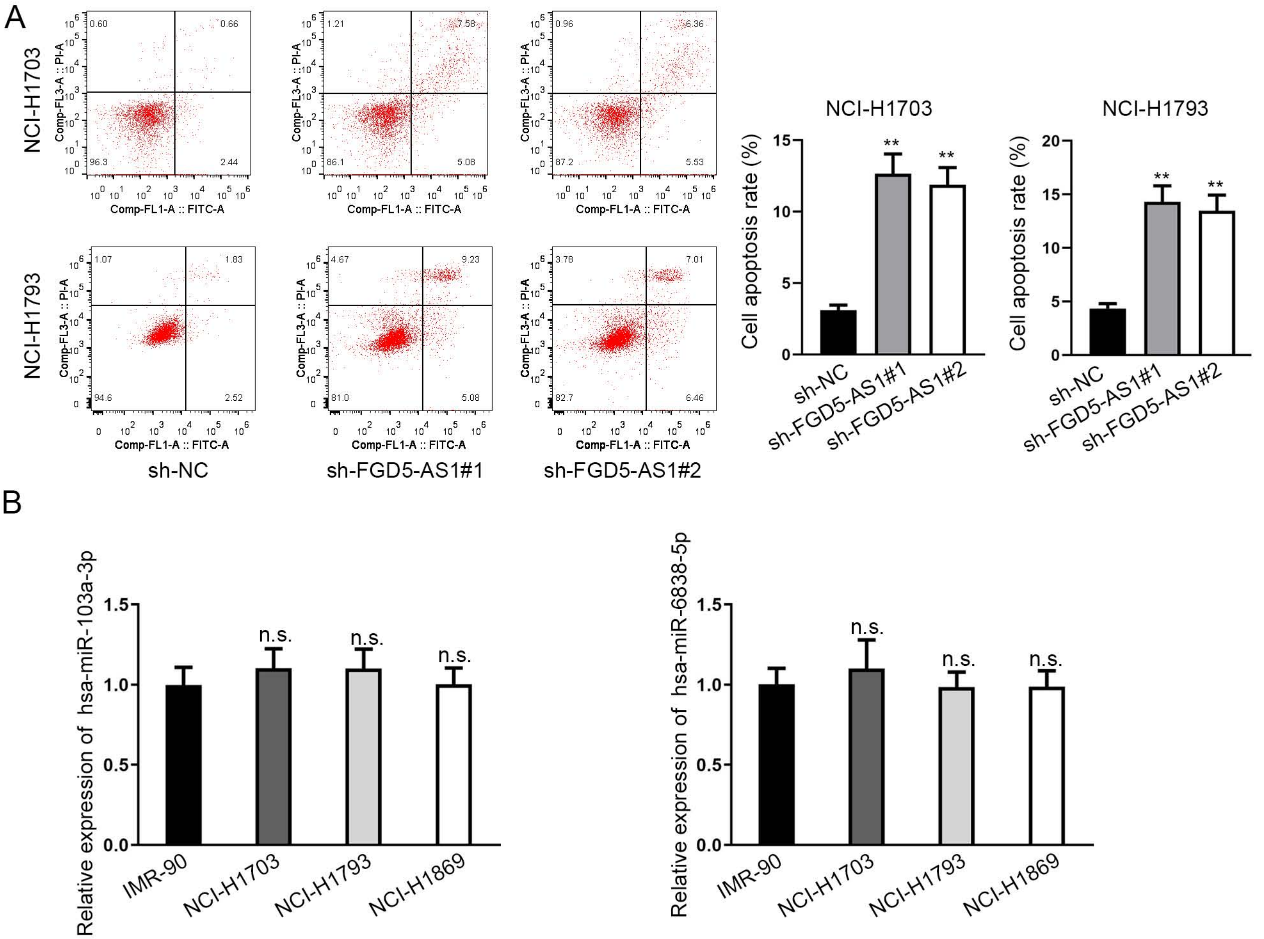
A
NCI-H1703
NCI-H1793
Comp-FL3-A :: PI-A
Comp-FL1-A :: FITC-A
0.60
0.66
96.3
2.44
1.21
7.58
86.1
5.08
0.96
6.36
87.2
5.53
1.07
1.83
94.6
2.52
4.67
9.23
81.0
5.08
3.78
7.01
82.7
6.46
sh-NC
sh-FGD5-AS1#1
sh-FGD5-AS1#2
NCI-H1703
NCI-H1793
Cell apoptosis rate (%)
**
B
Relative expression of hsa-miR-103a-3p
Relative expression of hsa-miR-6838-5p
n.s.
IMR-90
NCI-H1703
NCI-H1793
NCI-H1869

Figure S1(A) Flow cytometry analysis of FGD5-AS1-downregulated NSCLC cells. (B) The expression comparison of two miRNAs in one normal cell or three NSCLC cells. $^{**}P< 0.01$ indicated statistically significantdifferences in this figure. n.s.: no significance.

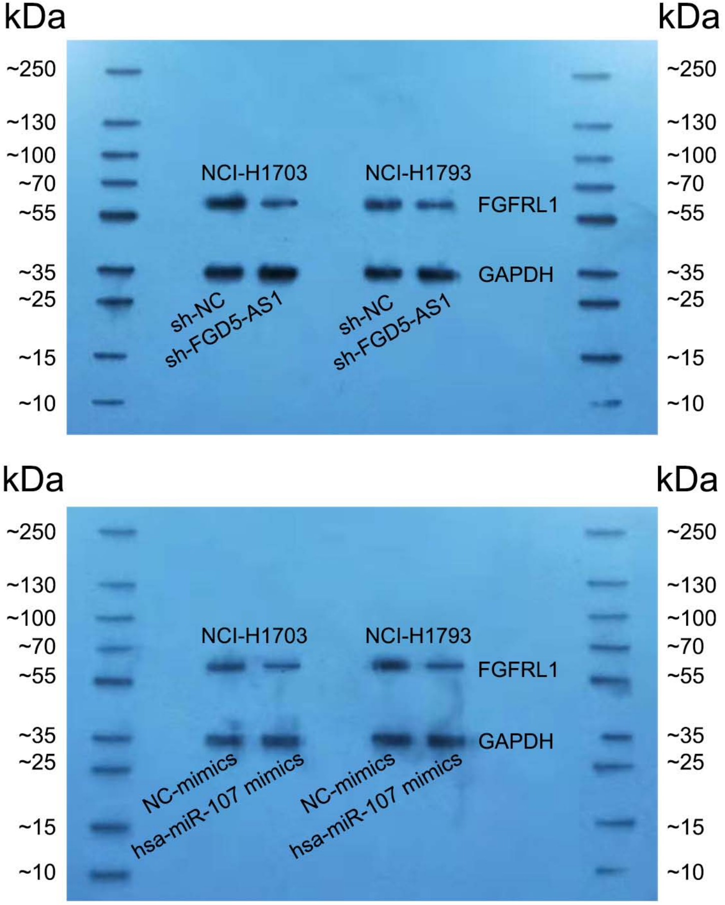
kDa
kDa
~250
~130
~100
~70
~55
~35
~25
~15
~10
NCI-H1703
NCI-H1793
FGFRL1
GAPDH
sh-NC
sh-FGD5-AS1
sh-NC
sh-FGD5-AS1
kDa
kDa
~250
~130
~100
~70
~55
~35
~25
~15
~10
NCI-H1703
NCI-H1793
FGFRL1
GAPDH
NC-mimics
hsa-miR-107 mimics
NC-mimics
hsa-miR-107 mimics

Table S1 Sequences used for FGFRL1 overexpression.

ATGACGCCGAGCCCCCTGTTGCTGCTCCTGCTGCCGCCGCTGCTGCTGGGGGCCTTCCCGCCG
GCCGCCGCCGCCCGAGGCCCCCCAAAGATGGCGGACAAGGTGGTCCCACGGCAGGTGGCCCG
GCTGGGCCGCACTGTGCGGCTGCAGTGCCCAGTGGAGGGGGACCCGCCGCCGCTGACCATGT
GGACCAAGGATGGCCGCACCATCCACAGCGGCTGGAGCCGCTTCCGCGTGCTGCCGCAGGGG
CTGAAGGTGAAGCAGGTGGAGCGGGAGGATGCCGGCGTGTACGTGTGCAAGGCCACCAACGG
CTTCGGCAGCCTGAGCGTCAACTACACCCTCGTCGTGCTGGATGACATTAGCCCAGGGAAGGA
GAGCCTGGGGCCCGACAGCTCCTCTGGGGGTCAAGAGGACCCCGCCAGCCAGCAGTGGGCAC
GACCGCGCTTCACACAGCCCTCCAAGATGAGGCGCCGGGTGATCGCACGGCCCGTGGGTAGCT
CCGTGCGGCTCAAGTGCGTGGCCAGCGGGCACCCTCGGCCCGACATCACGTGGATGAAGGAC
GACCAGGCCTTGACGCGCCCAGAGGCCGCTGAGCCCAGGAAGAAGAAGTGGACACTGAGCC
TGAAGAACCTGCGGCCGGAGGACAGCGGCAAATACACCTGCCGCGTGTCGAACCGCGCGGGC
GCCATCAACGCCACCTACAAGGTGGATGTGATCCAGCGGACCCGTTCCAAGCCCGTGCTCACA
GGCACGCACCCCGTGAACACGACGGTGGACTTCGGGGGGACCACGTCCTTCCAGTGCAAGGT
GCGCAGCGACGTGAAGCCGGTGATCCAGTGGCTGAAGCGCGTGGAGTACGGCGCCGAGGGCC
GCCACAACTCCACCATCGATGTGGGCGGCCAGAAGTTTGTGGTGCTGCCCACGGGTGACGTGT
GGTCGCGGCCCGACGGCTCCTACCTCAATAAGCTGCTCATCACCCGTGCCCGCCAGGACGATG
CGGGCATGTACATCTGCCTTGGCGCCAACACCATGGGCTACAGCTTCCGCAGCGCCTTCCTCAC
CGTGCTGCCAGACCCAAAACCGCCAGGGCCACCTGTGGCCTCCTCGTCCTCGGCCACTAGCCT
GCCGTGGCCCGTGGTCATCGGCATCCCAGCCGGCGCTGTCTTCATCCTGGGCACCCTGCTCCTG
TGGCTTTGCCAGGCCCAGAAGAAGCCGTGCACCCCCGCGCCTGCCCCTCCCCTGCCTGGGCAC
CGCCCGCCGGGGACGGCCCGCGACCGCAGCGGAGACAAGGACCTTCCCTCGTTGGCCGCCCT
CAGCGCTGGCCCTGGTGTGGGGCTGTGTGAGGAGCATGGGTCTCCGGCAGCCCCCCAGCACTT
ACTGGGCCCAGGCCCAGTTGCTGGCCCTAAGTTGTACCCCAAACTCTACACAGACATCCACAC
ACACACACACACACACTCTCACACACACTCACACGTGGAGGGCAAGGTCCACCAGCACATCC
ACTATCAGTGC
